# BPA and BPS Affect Connexin 37 in Bovine Cumulus Cells

**DOI:** 10.3390/genes12020321

**Published:** 2021-02-23

**Authors:** Reem Sabry, Charlotte Apps, Jaqueline A. Reiter-Saunders, Angela C. Saleh, Sumetha Balachandran, Elizabeth J. St. John, Laura A. Favetta

**Affiliations:** Reproductive Health and Biotechnology Laboratory, Department of Biomedical Sciences, Ontario Veterinary College, University of Guelph, 50 Stone Road East, Guelph, ON N1G 2W1, Canada; rsabry@uoguelph.ca (R.S.); capps@uoguelph.ca (C.A.); reitersaundersj@gmail.com (J.A.R.-S.); asaleh01@uoguelph.ca (A.C.S.); sbalacha@uoguelph.ca (S.B.); estjohn@uoguelph.ca (E.J.S.J.)

**Keywords:** Bisphenol A, Bisphenol S, connexins, oocytes, cumulus cells

## Abstract

Bisphenol S (BPS) is used as an alternative plasticizer to Bisphenol A (BPA), despite limited knowledge of potential adverse effects. BPA exhibits endocrine disrupting effects during development. This article focuses on the impact of bisphenols during oocyte maturation. Connexins (Cx) are gap junctional proteins that may be affected by bisphenols, providing insight into their mechanism during development. Cxs 37 and 43 are crucial in facilitating cell communication between cumulus cells and oocytes. Cumulus-oocyte complexes (COCs), denuded oocytes, and cumulus cells were exposed to 0.05 mg/mL BPA or BPS for 24 h. Both compounds had no effect on Cx43. Cumulus cells exhibited a significant increase in Cx37 expression following BPA (*p* = 0.001) and BPS (*p* = 0.017) exposure. COCs treated with BPA had increased Cx37 protein expression, whilst BPS showed no effects, suggesting BPA and BPS act through different mechanisms. Experiments conducted in in vitro cultured cumulus cells, obtained by stripping germinal vesicle oocytes, showed significantly increased expression of Cx37 in BPA, but not the BPS, treated group. BPA significantly increased Cx37 protein expression, while BPS did not. Disrupted Cx37 following BPA exposure provides an indication of possible effects of bisphenols on connexins during the early stages of development.

## 1. Introduction

Endocrine disrupting compounds (EDCs) are defined as exogenous agents that can act as either receptor agonists or antagonists. EDCs interfere with hormone synthesis, secretion, transport and elimination, thereby affecting metabolism, hormonal homeostasis, and developmental processes in the body [1]. Bisphenol A (BPA), which imbalances hormone-driven processes in both humans and animals, is one of the most environmentally prevalent EDCs. BPA is a synthetic, xenobiotic, organic compound widely used as a plasticizer in polymeric plastics, particularly polycarbonate plastics and epoxy resins [1]. These polymers are linked by ester bonds that, upon exposure to high temperatures, acidic or basic substances and ultraviolet rays, can hydrolyze, causing BPA to leach from the plastic into the environment [2]. As a result, BPA has been detected in the air, water, and soil [3,4]. Its long half-life and lipophilic characteristics have led to bioaccumulation in several species including humans [5] and farm animals. While the effects of EDCs are well-studied in humans, wild and farm animals are also exposed to relatively high concentrations of EDCs because they persist in the environment and concentrate in fat tissue becoming released when fat is mobilized during pregnancy or lactation, thus exposing embryos and neonates to relatively high concentrations. EDC rates of ingestion and metabolism in farm animals have not been widely studied, but there is clearly a potential risk of significant bioaccumulation and its associated effects on the health and reproductive capacity [6]. Feed and water, packaged in plastic containers, are often considered to be the most important routes of exposure of farm animals to most EDCs, including bisphenols [6].

BPA disrupts the normal function of various cell signaling pathways through its ability to mimic estrogen, by binding to numerous receptors including the estrogen receptors [3,7]. BPA exposure has been linked to many reproductive disorders, including recurrent miscarriage, reduced fertilization and abnormal oocyte meiotic maturation [8]. In vitro BPA exposure also reduced cumulus cell proliferation, and increased oocyte degeneration and spindle/chromosome abnormalities [1,4]. Previous studies from our group have shown significantly decreased cleavage and blastocyst rates upon exposure to BPA as well as extensive changes in gene expression in the bovine species [9,10,11]. Additionally, BPA can also affect the steroidogenic activities and proliferation of granulosa cells surrounding the oocyte thereby interrupting oocyte maturation through an additional mechanism of action [12]. It is due to these widespread adverse effects that BPA has been largely eliminated from plastics manufacturing and replaced by alternatives [13]. Bisphenol S (BPS) is now a widespread alternative, despite its potential negative reproductive or endocrine disrupting effects being unclear [14]. For instance, a study that considered the US and seven Asian countries detected BPS in approximately 81% of urine samples [15].

Although BPS does not leach as easily as BPA, it has been found to have similar hormone-mimicking effects by demonstrating estrogenic and antiandrogenic activity [13,16,17]. In vivo, BPS exposure led to embryo malformation, decreased hatching, thyroid hormone and estradiol disruption, and altered gene expression in male and female zebrafish [18]. BPS also altered the embryonic development rates and gene expression in zebrafish [19]. Moreover, BPS exposure disrupted oocyte maturation, decreased cumulus cell expansion and induced cytoskeletal disruption in pig oocytes [20]. Lastly, in more recent studies, BPS has also been reported to alter steroidogenic genes and pathways in sheep and human granulosa cells with an overall reduction in progesterone and estrogen secretion and a decrease in oocytes that reached maturation [21,22]. BPS is used as substitute in plastics, but there is a lack of research supporting their safety [23]. BPS has structure similar to that of BPA [24], is found in BPA-free labeled products and has no identified tolerable dose intakes and is therefore currently unregulated [25,26].

A known effect of both BPA and BPS is disruption to cumulus cell expansion, which is dependent on the oocyte-secreted proteins, such as GDF-9. Bisphenols may be causing interference by altering the bidirectional signaling between oocytes and their cumulus cells [4,27]. Given that this crosstalk regulates oocyte maturation and meiotic cycles, the disrupted communication could have downstream effects and account for the improper meiotic cycles and decreased oocyte quality, reported in the aforementioned studies. This intercommunication between the oocyte and its surrounding cumulus cells, is known as gap junctional intercellular communication (GJIC) and occurs via specialized gap junction proteins, or connexons. Connexons are channels made up of clusters of connexin molecules that aggregate to form a functioning gap junction between two neighboring cells [4]. These integral membrane proteins link the cytoplasm of neighboring cells to allow exchange and transport of molecules via passive diffusion [4,28]. Through knockout experiments in mice, connexins have been shown to be essential for oogenesis as well as folliculogenesis [29]. Mammalian cumulus-oocyte-complexes (COCs) express multiple connexins and utilize them for the intercellular communication required for proper development [30], particularly pre-implantation development [31]. Gap junctions facilitate this communication between adjacent cells and provide the oocyte with essential nutrients and regulatory molecules [30,32]. Examples of these proteins include connexins 43 and 37, co-expressed in both human and bovine oocytes. Localization of Cx37 and Cx43 varies between species, but there is evidence in the literature showing that Cx37 is concentrated mainly on the oocyte surface in many species (murine, ovine, bovine) and is largely involved in oocyte-cumulus cell cross-talk [32,33]. In contrast, Cx43 functions mainly to couple adjacent cumulus cells and aid in their communication [30,32]. In the bovine species, Cx43 knockout demonstrates its importance for cell compaction and embryo development, while in humans, it mediates embryonic pluripotency [31,34]. Studies in mice have demonstrated that Cx37 is specifically required for ovarian function. A 2010 study showed that oocytes retrieved from Cx37- knockout mice were unable to reach meiotic competence due to the loss of metabolic coupling with surrounding cumulus cells [35,36].

Hormones also play a role in gap junctional communication between cumulus cells, facilitating cumulus expansion around the oocyte [32]. The goal of this study was to address the effects of BPA and BPS, at the current Lowest Observed Adverse Effect Level (LOAEL) dose (0.05 mg/mL) [37,38], on both Cx43 and 37 during in vitro maturation (IVM) of bovine cumulus-oocyte-complexes (COCs), as well as in vitro culture (IVC) of bovine cumulus cells. This is the lowest dose at which an adverse effect is observed in humans and was previously shown to have significant adverse effects on bovine pre-implantation development, while being environmentally and physiologically important [38]. Our group previously performed a WST assay [38] and found that BPA at the LOAEL dose significantly decreased cell viability, while BPS did not. We have also performed a dose dependent assay where doses lower and higher than the LOAEL were tested on cell viability, with this dose found to be the optimal exposure dose that is both environmentally and physiologically relevant. The developmental rates for blastocysts treated with the LOAEL dose of BPA and BPS during bovine oocyte maturation were previously calculated by our group, where a significant decrease in both cleavage and blastocyst rates were consistent with BPA, but not BPS, exposure [38]. This study creates the basis of a possible alternative mechanism of action of bisphenols during early development in addition to the most studied action on the estrogen receptors. This research is in-line with several other studies from our group showing that bisphenols can act through different receptors [11] and/or affecting epigenetics changes through alterations of specific microRNAs [38], aiming to find alternative mechanisms of action of bisphenols to explain their significant effects in mammalian organisms during early development.

## 2. Materials and Methods

### 2.1. Oocyte Collection and Maturation

Bovine ovaries were obtained from a local slaughterhouse (Cargill Meat Solutions Distribution; Guelph, ON, Canada) and follicles were aspirated following the protocol outlined by Ferris et al. [10]. The aspirated COCs were collected into 1M HEPES buffered F-10 Ham (Sigma Aldrich; Oakville, ON, Canada) nutrient mixture containing 2% fetal bovine serum (FBS) (GIBCO; Whitby, ON, Canada), Heparin (2 IU/mL, Sigma Aldrich; Oakville, ON, Canada), and penicillin/ streptomycin (10,000 IU/mL/ 10,000 IU/mL, Invitrogen; Burlington, ON, Canada). The base of the treatment groups was a maturation media: 10mL SAGE in vitro maturation (S-IVM, TCM-199) media (Caisson Labs; Smithfield, UT, USA), with 800 µL FBS, sodium pyruvate (Sigma-Aldrich; Oakville, ON, Canada), 0.2M L-glutamine (Sigma Aldrich; St. Louis, MO, USA) and 0.6% penicillin/streptomycin (Invitrogen; Burlington, ON, Canada). The following hormones (H) were also added: 2.5 µg/mL follicle-stimulating hormone (FSH—Folltropin) (Vetoquino; Cambridge, ON, Canada), 1 µg/mL estradiol, and 1 µg/mL luteinizing hormone (LH) (NIH; San Diego, CA, USA). The BPA and BPS (Sigma Aldrich; Oakville, ON, Canada) stock solutions were generated in 0.1% ethanol and stored in glass vials. Droplets of four treatment groups were created: control (containing only S-IVM+H), vehicle (containing S-IVM+H with 0.1% ethanol), BPA and BPS groups, each with their respective chemicals, dissolved in 0.1% ethanol, added to the S-IVM+H at a concentration of 0.05 mg/mL. After 24 h maturation, oocytes from each of the treatment groups were imaged under a stereomicroscope at 9X objective. For RNA extraction, 60 COCs were collected and matured for 24 h in 38.5 °C, 5% CO_2_ incubator. COCs were then washed with PVA/PBS buffers and divided into two groups of 30. One group contained intact COCs and the other group were denuded in order to investigate oocytes and cumulus cells seperatley and all samples kept at −80 °C for future use. For protein quantification, the sample size was increased to 40 COCs, 40 oocytes, and cumulus cells denuded from 40 oocytes to obtain enough proteins to be detected by Western blotting.

### 2.2. In Vitro Fertilization (IVF)

*Bos Taurus* semen from Semex (Guelph, ON, Canada) was thawed and prepared for swim-up in 1.5 mL of HEPES/sperm TALP, with 1 mL bovine serum albumin (BSA) added. Samples were placed in the incubator at 38.5 °C, 5% CO_2_, for 45 min. 24 h after maturation under the four different treatment conditions stated above (Control, Vehicle, BPA, and BPS), oocytes were washed twice in HEPES Sperm TALP followed by 20 µg/mL heparin in IVF TALP and placed into 80 µL IVF TALP droplets in mineral oil (Sigma Aldrich, St. Louis, MO, USA). Motile sperm were collected and spun for 7 min at 200× *g*. After which, droplets containing twenty oocytes received 1 × 10^6^ sperm cells/mL/drop were kept in 5% CO_2_ at 38.5 °C for 24 h. After 18 h, presumptive zygotes were stripped of cumulus by vortexing and washed twice in both HEPES/sperm TALP and synthetic oviductal fluid (SOF) media containing sodium pyruvate, essential and non-essential amino acids, 15% BSA, gentamicin, and FBS. Zygotes were then transferred to 30 μL SOF droplets covered with mineral oil and were incubated at 38.5 °C, 5% O_2_ until day 8 blastocyst stage for all treatment groups.

### 2.3. Cell Culture and Treatment

COCs were aspirated from follicles using the same methods as previously outlined. Approximately 100–200 COCs were stripped of their cumulus cells using mechanical disruptions via a micropipette. Cumulus cells were placed in 15 mL conical tubes containing 8 mL of 1× Dulbecco’s Modified Eagle Medium (DMEM) (Gibco) containing glutamine (2 mM) (Sigma-Aldrich) and penicillin/streptomycin (1%). Cells were resuspended in DMEM supplemented with 20% FBS, plated on a T25 flask or T75 flask (Corning) for RNA and protein extraction, respectively, and cultured at 38.5 °C in 5% CO_2_ for 6–7 days with media replacement every 48 h until no empty patches are observed. At 100% confluency, the cells were passaged twice, split at passage 2 into 4 flasks using DMEM +10% FBS, and incubated. After 12 and 30 h for T25 and T75, respectively, the cells were serum restricted with DMEM supplemented with 0.1% FBS. 12 and 18 h (T25 and T75 respectively) post serum restriction, flasks were randomly assigned to a group and treated using the same doses and groups as above but under different media conditions: DMEM + 0.1% FBS (Control), 0.1% ethanol in DMEM + 0.1% FBS (Vehicle), BPA in DMEM + 0.1% FBS (BPA—0.05 mg/mL), and BPS in DMEM + 0.1% FBS (BPS—0.05 mg/mL). A total of 24 h after treatment, cells were trypsinized, washed in PBS/PVA, snap frozen in liquid nitrogen, and stored at −80 °C. A total of 24 h after treatment, cumulus cells were imaged under an inverted phase contrast microscope at 10× objective.

### 2.4. RNA Extraction

RNA was later extracted from in vitro matured COCs, oocytes, and cumulus cells as well as from in vitro cultured cumulus cells using RNeasy Micro kits (QIAGEN; Toronto, ON, Canada), according to the manufacturer protocol [10]. RLT Plus buffer, 70% ethanol, RW1 buffer, RPE buffer, and 80% ethanol were used for separation, in that order. All samples were reverse transcribed: 1 µg RNA from IVM COC and cumulus cells, 100 ng RNA from IVC cumulus cells, whereas for oocytes, 30 cells were consistently reverse transcribed using the Quantabio qScript cDNA supermix (VWR, Mississauga, ON, Canada), as outlined by the manufacturer, under the following reaction protocol: 5 min at 25 °C, 30 min at 42 °C, and 5 min at 85 °C and the resulting cDNA stored at −20 °C. 

### 2.5. Quantitative Real-Time Polymerase Chain Reaction (qPCR)

SsoFast Evagreen Supermix (Bio-Rad; Mississuaga, ON, Canada) was used for target specificity and fluorescence amplitude for target quantification on the qPCR. Experiments were conducted on three biological replicates and 3 technical replicates were used for each biological replicate to account for errors. Glyceraldehyde-3- phosphate dehydrogenase (*GAPDH*) and Peptidylprolyl isomerase A (*PPIA*) were used as housekeeping genes [10] at a final primer concentration of 0.5 µM (Sigma Aldrich) for in vitro matured (IVM) COCs, oocytes, and cumulus cells. Specific primers for Cx37 and Cx43 were used at a final concentration of 0.5 µM (Sigma Aldrich; Oakville, ON, Canada) as well. For in vitro cultured cumulus cells (IVC), GAPDH and PPIA were used at a concentration of 25 nM and Cx37 at 10 nM. Primers information are listed in Table 1. There was no significant effect on Cx43 in the oocytes, so it was removed from further analysis and was not investigated in the cumulus cell culture experiments. The two reference genes were previously confirmed as the most stable genes for this treatment, according to a GeNorm analysis [38]. The PCR protocol was carried out as follows: denaturation at 95 °C for 10 s, annealing at 60 °C for 10 secs, and extension at 72 °C for 20 secs for a total of 40 cycles with a melt temperature analysis at the end of each cycle (Bio-Rad CFX Manager 3.1). The data was normalized using the ΔΔCt method and primer efficiencies were calculated in both IVM and IVC samples using a standard curve method for each primer and are listed in Table 1 below. All quantifications were done on at least three biological replicates in technical triplicates.

### 2.6. Western Blotting

Protein was extracted from 40 COCs per treatment group for a total of 160 COCs by RIPA lysis buffer (containing added protease inhibitors), subsequent freeze-thaw cycles (frozen in liquid nitrogen; thawed on ice), and final sonication in an ice-water bath for 30 min, followed by centrifugation at 13,800 g at 4 °C for 10 min. Extracted proteins were quantified using a Bradford Assay (Bio-Rad; Mississauga, ON, Canada) in order to load 30 µg of protein for IVM COCs, oocytes, and cumulus cells and IVC cumulus cells, respectively. A 3× reducing buffer was used, with added 2-mercaptoethanol to a final concentration of 12M.

Samples were run on a 12% SDS PAGE agarose gel at 125V for 1.5 h, in an Invitrogen Novex apparatus. Gels were made with acrylamide (BioRad, 500 mL 30% acrylamide, cat# 1610156). A BioRad Precision Plus Dual Color Standards molecular weight marker (#1610374, Abcam; Toronto, Canada) was loaded in lane 1 at a volume of 12 µL. An MCF7 whole cell lysate (ab3871, 2 µg/µL; Abcam; Toronto, Canada) was selected as a positive control, and loaded at a volume of 5 µL and 10 µL for IVM and IVC samples, respectively. A larger volume of MCF7 was used in IVC cumulus cells due to the higher concentrations of proteins loaded (30 µg). Protein samples were loaded after being denatured by boiling them for 6 min. Gel was transferred to a Nitrocellulose membrane, following a Wet Transfer protocol, at 25V for 2.5 h in 1× transfer buffer. After the wet transfer, the membrane was washed in 1× TBST and then blocked in a 5% skim milk 1× TBST solution for 1 h. The primary antibody specific for Cx37 (ab181701 GJA4 N-terminal; abcam; Toronto, Canada), diluted 1:1000, was incubated with the membrane overnight at 4 °C. 3 × 10 min washes in 1× TBST followed and incubation with the anti-rabbit secondary antibody (Cell Signaling Technology 7074S; Denver, MA, USA), diluted 1:5000, was carried out for 1 h at room temperature. After the hour, the membrane was once again washed with 1× TBST (3 × 10 min).

The membrane was coated with a chemiluminescence substrate (BioRad; Mississauga, ON, Canada), containing equal parts oxidizing and luminal solutions, for 5 min and then imaged in the BioRad ChemiDoc. After imaging, a 1:10000 anti-α-tubulin primary antibody (ab7291, abcam; Toronto, Canada) dilution, which was made using 10 mL of the skim milk blocking solution, was added to the membrane for 1 h as a loading control. After washing with TBST, 10mL of 1:5000 anti-mouse secondary antibody (Cell Signaling Technology 7076S; Denver, MA, USA) dilution, made using the blocking solution, was added for 1 h. After washing the membrane with TBST, the membrane was imaged again. Densitometric analysis was carried out using the ImageLab software on at least three biological replicates.

### 2.7. Confocal Microscopy

COCs were washed twice in PBS+PVA, fixed for 1 h in 4% Paraformaldehyde (PFA) and stored in 2% PFA until the time of use. A total of five samples per treatment group were processed at a time, using a 60-well MicroWell MiniTray (M0815; Sigma-Aldrich). Samples were first blocked for 1 h at room temperature in 1X PBS + 0.01% Triton X100 + 5% NDS, then transferred to a 1× PBS wash for 20 min in a dark, humidified chamber at 38.5 °C. Samples incubated with the Cx37 primary antibody (ab181701 GJA4 N-terminal; abcam; Toronto, Canada) diluted with antibody dilution buffer (1:100) in 1× PBS + 0.01% Triton X100 + 5% NDS, while the negative controls were incubated in 1× PBS only overnight at 4 °C in a dark, humidified chamber. After 3 × 30 min washes in an antibody dilution buffer at 38.5 °C in a dark, humidified chamber, samples were placed in the secondary antibody, donkey Anti-Rabbit IgG H&L Alexa Fluor 488 (#A-21206; Invitrogen, ThermoFisher Scientific; Mississuaga, ON, Canada), diluted with antibody dilution buffer (1:200), for 1 h at 4 °C in a dark, humidified chamber and then transferred to Hoechst stain (H33342; ThermoFisher Scientific; Mississuaga, ON, Canada) for 30 min at 38.5 °C in a dark, humidified chamber. Finally, samples were washed 2 × 30 min in an antibody dilution buffer at 38.5 °C in a dark, humidified chamber and mounted on slides with Vectashield (Vector Laboratories, Inc.; Burlingame, CA, USA). Slides were stored at 4 °C and read within one week of preparation. Slides were imaged on the Olympus FV1200 Confocal Microscope, using the LD laser for visible light (at 405 nm) and Multiline Argon Laser (488 nm), at 20X and 40X dry objectives.

### 2.8. Statistical Analysis

GraphPad Prism 6 software was used to analyze the statistical difference amongst the treatment groups. Each data set was tested for normality using Kolmogorov–Smirnov and Shapiro Wilk tests. Normally distributed data sets were analyzed using one-way analysis of variance (ANOVA) and not normally distributed data sets were analyzed using Kruskal-Wallis test. Differences at a two-tailed *p*-value < 0.05 were considered statistically significant. Data sets with a statistically significant *p* value were then subjected to Tukey’s post-hoc test in order to compare differences between each treatment group. These tests were also used to analyze protein expression values obtained from densitometry analysis of western blots relative to the loading control, α-tubulin (Cell signaling Technology). Data shown represent the mean +/− standard error of the mean (SEM).

## 3. Results

### 3.1. Oocyte and Embryo Morphology

**Figure 1:**
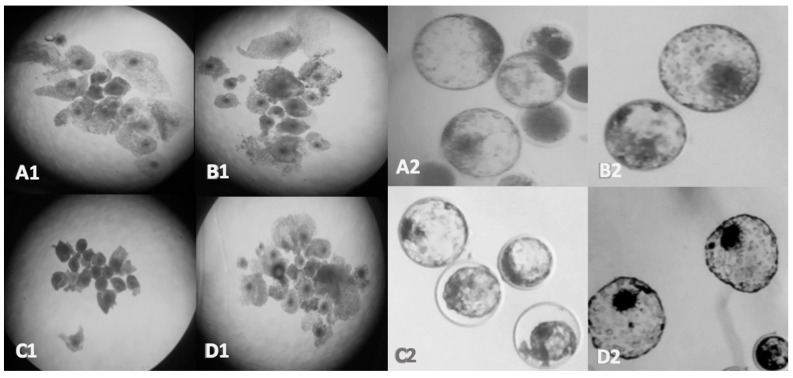
Morphological features of Bos Taurus COCs and embryos. Oocyte maturation after 24 hrs (**left**) and day 8 embryo development (**right**); (**A**): Control, (**B**): Vehicle (0.1% ethanol), (**C**): BPA (0.05 mg/mL), (**D**): BPS (0.05 mg/mL) imaged using a 10× objective for the oocytes and at 40× objective for the blastocysts.

Figure 1 depicts COCs 24 hr post maturation using a stereomicroscope, highlighting the apparent morphological changes in oocytes of the BPA group. The number of blastocysts compared per condition was dependent on the treatment since blastocyst yields are decreased in the BPA group. Overall, the average number of blastocysts analyzed between three biological replicates are 12 for the control, 10 for the vehicle, 5 for the BPA groups, and 11 for the BPS groups. They appear small and dark indicating failure to undergo cumulus cell expansion (Figure 1C1). Blastocysts in the BPA group present observable characteristics that suggest low quality embryos including a collapse in the inner cell mass (ICM), fewer expanded embryos, improper trochectoderm organization, and ICMs with fewer cells, seen in Figure 1C2. Overall, embryos in the BPA group do not possess the adequate features normally observed by day 8 and this is indicative of a poor prognosis for developmental success [41]. BPS exposure caused no change in the morphology of the COCs, but an accelerated embryo development with hatched blastocysts observed by day 8 (Figure 1D2).

### 3.2. Cell Culture Treatments

**Figure 2:**
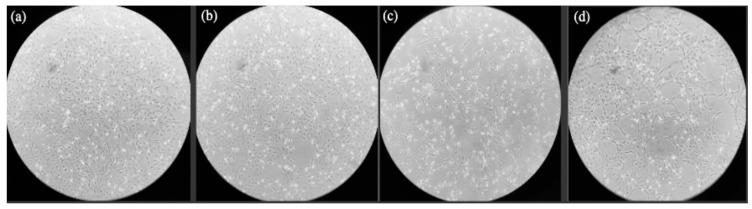
Morphological features of Bos Taurus cumulus cells. (**a**) Control group after 24-hr treatment; (**b**) Vehicle group after 24-hr treatment; (**c**) BPA group after 24-hr treatment; (**d**) BPS group after 24-hr treatment. Images obtained by inverted phase contrast microscope at 10× objective.

After the 24-h treatment period, the control, vehicle, and BPS groups all had similar levels of confluency at an estimated confluency of 90–100% for the control and vehicle groups and a 60–70% confluency for the BPS group, as shown in Figure 2a,b,d, respectively, after imaging with an inverted phase contrast microscope. Conversely, the BPA-treated group was estimated to be at 40–50% confluency, Figure 2c.

### 3.3. Cx43 and Cx37 Expression in COCs, Oocytes, and Cumulus Cells

**Figure 3:**
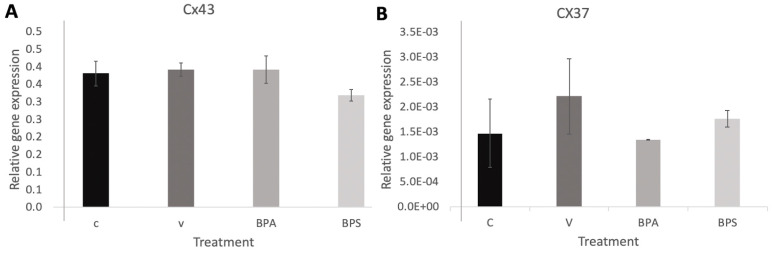
Connexin mRNA expression in bovine COCs. (**A**) CX43 and (**B**) Cx37mRNA expression in bovine COCs after 24 h of maturation in S-IVM (C), S-IVM and 0.1% ethanol (V), S-IVM and 0.1% 0.05mg/mL (BPA), S-IVM 0.1% 0.05mg/mL (BPS), *N* = 3.

**Figure 4:**
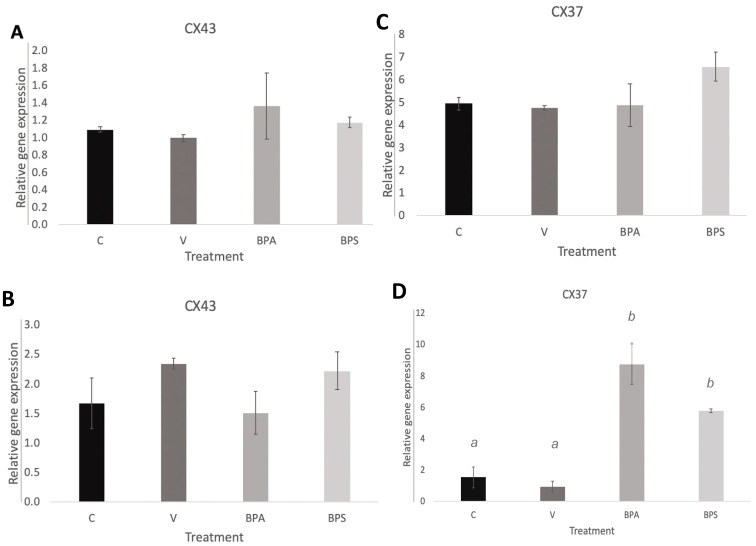
Connexin mRNA expression in bovine oocytes and cumulus cells. CX43 in oocytes (**A**) and in cumulus cells (**B**) as well as Cx37 in oocytes (**C**) and in cumulus cells (**D**) mRNA after 24 h of maturation in S-IVM (C), S-IVM and 0.1% ethanol (V), S-IVM and 0.1% 0.05mg/mL (BPA), S-IVM 0.1% 0.05 mg/mL (BPS). Letters indicates statistical significance at *p* < 0.05, BPA (*p* = 0.001) and BPS (*p* = 0.017) and *n* = 3.

No significance difference was detected for Cx43 (Figure 3A) nor Cx37 (Figure 3B) in COCs. In addition, no significant changes in Cx43 mRNA expression were detected in neither the oocyte (Figure 4A) nor cumulus samples (Figure 4B). No changes in Cx37 mRNA expression were measured in denuded oocytes (Figure 4C), but a significant increase in Cx37 mRNA expression in cumulus cells after exposure to both BPA (*p* = 0.001) and BPS (*p* = 0.017) was observed (Figure 4D). This suggests that bisphenols may affect connexin activity and their role in communication between cumulus cells, disrupting the environment surrounding the maturing oocyte. The error bars in the graphs represent three biological replicates with each biological replicate representing 30 COCs per treatment group for a total of 90 COCs per treatment group.

While we were unable to quantify levels of Cx37 protein in denuded oocytes or their corresponding cumulus cells, we were able to quantify protein levels in COCs. A total of three biological replicates were used for this analysis. These results support a possible role of Cx37 in facilitating oocyte-cumulus cell communication at the surface of the oocyte. When the COC complex is intact, the Cx37 between oocyte and cumulus cells is intact and able to be detected, while physical stripping of the cumulus cells could induce lack of detection at the no longer existing interface oocyte-cumulus cells.

**Figure 5:**
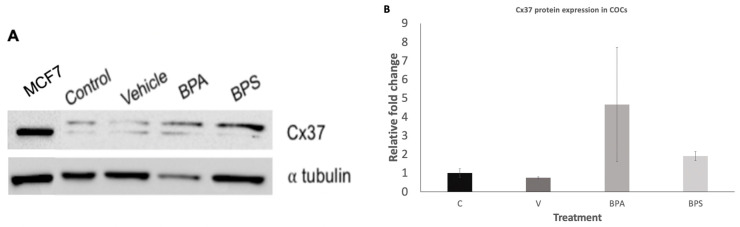
Connexin protein expression in bovine COCs. (**A**) Western Blot showing protein expression of Cx37 after 24-h maturation in SIVM (control), S-IVM with 0.1% ethanol (vehicle), S-IVM + 0.1% 0.05 mg/mL BPA (BPA), and S-IVM + 0.1% 0.05 mg/mL BPS (BPS). a tubulin: loading control. (**B**) Densitometry analysis for 3 biological replicates, depicting the relative fold change of Cx37 protein expression for treatment groups as compared to the control. Results showed an 8.14-fold increase in BPA group. Letters indicates statistical significance at *p* < 0.05, BPA (*p* = 0.0418, *n* = 3).

Densitometry analysis performed on three biological replicates (*n* = 3), and their corresponding loading control, α tubulin (Figure 5A), revealed a significant increase in Cx37 in BPA treated COCs (*p* = 0.0418). Compared to the control group, BPA showed an 8.14-fold increase in Cx37 expression while no significant effect was seen in the BPS exposed group (Figure 5B).

### 3.4. Cx37 Protein Localization in COCs, Oocytes, and Cumulus Cells

**Figure 6:**
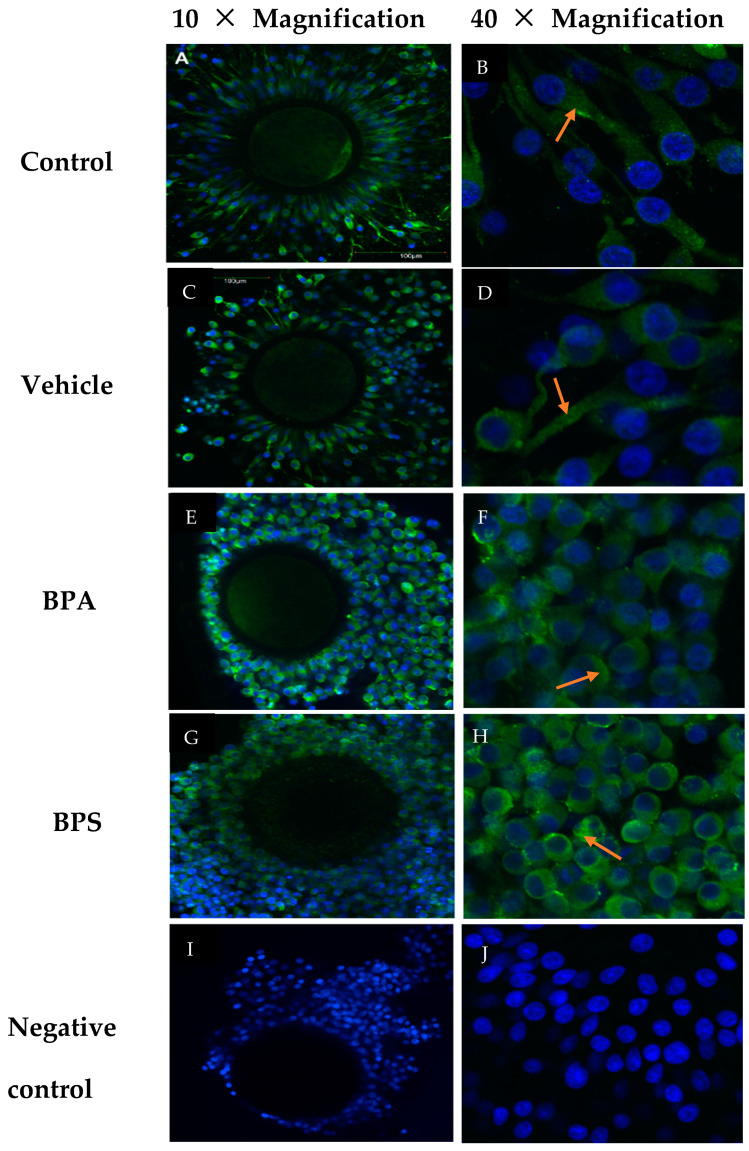
Confocal Microscopy of bovine COCs at 10× and 40× magnification. (**A**,**B**) S-IVM (control), (**C**,**D**) S-IVM with 0.1% ethanol (vehicle), (**E**,**F**) S-IVM + 0.1% 0.05 mg/mL BPA (BPA), and (**G**,**H**) Scheme 0. 0.05 mg/mL BPS (BPS). Blue: Hoechst (nucleic acid) stain, Green: Cx37 (Alexa Fluor 488) indicated by red arrows. (**I**,**J**) A negative control group was treated with PBS in place of 1°Ab at 40× with the arrows pointing to Cx37 localization within the cytoplasm.

As denuded oocytes showed no expression of Cx37, we analyzed only COCs by confocal microscopy to localize Cx37. As seen in Figure 6, there were no changes in protein localization between treatment groups. In all groups, Cx37 remained diffusely localized to the cytoplasms of the cumulus cells surrounding the oocyte. This is shown at two magnifications, 10X and 40X, where the higher magnification clearly depicts Cx37 labelling in the cytoplasm of cumulus cells. Imaging was done on 10 COCs per treatment group, and five negative control COCs.

### 3.5. Cx37 Expression in In Vitro Cultured Cumulus Cells

**Figure 7:**
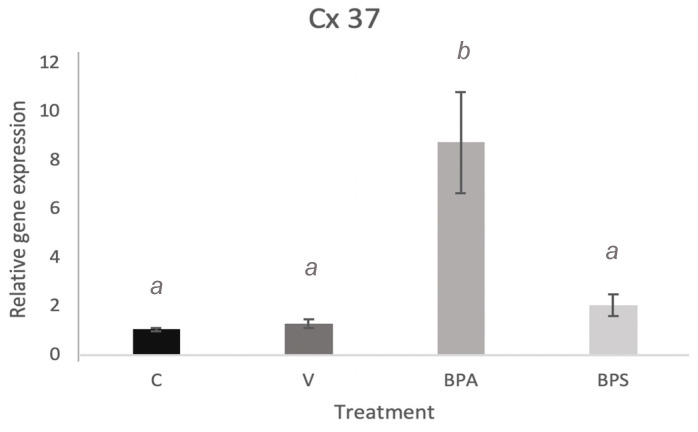
Connexin mRNA expression in bovine cumulus cells. Quantification of Cx37 mRNA after 24 h treatment in the four experimental groups: control, vehicle of 0.1% ethanol, 0.05 mg/mL BPA in 0.1% ethanol and 0.05 mg/mL BPS in 0.1% ethanol. Letters indicates statistical significance at *p* < 0.05, BPA (*p* < 0.001, *n* = 4).

**Figure 8:**
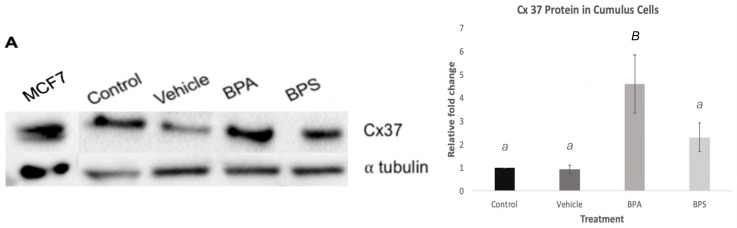
Connexin protein expression in bovine cumulus cells. (**A**) Blot showing protein expression of Cx37 after 24-h treatment with BPA and BPS (**B**) Densitometry analysis for 3 biological replicates, depicting the relative fold change of Cx37 protein expression for each treatment groups as compared to the controls. Letters indicates statistical significance at *p* < 0.05, BPA (*p* < 0.01, *n* = 6).

Quantification of Cx37 mRNA in cumulus cells shows a significant increase (*p* < 0.001) after BPA exposure (Figure 7). A densitometric analysis was performed to quantify protein levels of Cx37 in the different treatment groups. As shown in Figure 8A, BPA exposure had a statistically significant effect on Cx37 (*p* < 0.01) with an increase in protein expression, in comparison to both the control and vehicle group. Conversely, Cx37 protein expression in the BPS group did not show any statistically significant difference compared to the other three groups (Figure 8B).

## 4. Discussion

BPA effects are clearly visible morphologically at the matured oocyte stage and at the blastocyst stage, respectively. Darker, smaller, unexpanded COCs (Figure 1C1) are strong indicators of oocytes that have not undergone cumulus expansion, which is associated with negative fertility outcomes [42]. Correspondingly, day 8 blastocysts developing from oocytes treated with BPA analyzed under light microscopy appear to show higher levels of low quality characteristics than those in the control or vehicle groups. Although some blastocysts appear to be of excellent quality, some blastocysts have collapsed, while others have failed to expand (Figure 1C2). Day 8 blastocysts from BPS treated oocytes also appear to be good quality; suggesting that BPS does not affect development, like its predecessor, BPA.

Isolating/culturing cumulus cells is a valuable complement to our previous experiments since it provides a model where controlled manipulation of cellular functions is possible. In vitro culture of cumulus cells has been extensively utilized in endocrine research as early as 1989 in rats [43], with particular focus on the effects of reproductive toxicants on these cellular processes [12,22,44]. Cumulus cells are quickly being recognized for their importance as experimental models as they offer a non-invasive approach to oocyte quality assessment and prediction of embryonic fates [45]. In this study, bovine cumulus cells were studied in vitro in two ways: co-cultured with their oocyte in an in vitro maturation media for 24 h or separated from their oocytes and used to create primary cell cultures. These experiments present two different models to study a variety of cellular functions and processes within cumulus cells. In the former, cumulus cells are still connected to their oocytes via transzonal projections and gap junctions that closely mimics in vivo conditions and allows for the transport of crucial transcripts and substrates to prepare for maturation [46].

On the other hand, the strict requirements of maturation media and the short time of culture limits the possibilities for experimental manipulation and restricts studies to a single developmental stage, oocyte maturation. Therefore, various studies have adopted an alternative approach to culture cumulus cells separately over a longer period of time in order to study and compare specific features of cumulus cells, including gene expression profiles at various developmental stages [44,47,48,49]. In our study, cumulus cells were isolated before maturation in the absence of IVM hormones (LH, FSH, and estradiol) and without communication to the oocyte. The isolation of cumulus cells from their environment and subsequent attachment to culture flasks has been shown to induce differentiation and cellular stress [50,51]. The use of early passages and serum free media conditions retain some characteristics of the originating cells [50]; however, the connexin pathways between these attached cells and their separated counterparts may differ. Therefore, it is crucial to validate any findings in the cultured cumulus cells to findings in their counterparts.

When cumulus cells were isolated, cultured in vitro, and exposed to BPA and BPS, similar effects on cell morphology and time of division to reach confluency were observed under light microscopy. The control and vehicle groups contained round, elevated cells with 90–100% flask confluency. In contrast to this, both BPA and BPS treated cells had flattened filamentous cells with distinct cytoplasmic extensions and an overall lower flask confluency (Figure 2). Similar effects were seen in BPA treated human cumulus cells in a study by Mensur et al. [52]. This is usually an indication of higher levels of apoptosis or cell death after BPA exposure and is consistent with previous studies by our group, showing that BPA increases expression of apoptotic genes in bovine oocytes [10,53]. Morphology alone is an observational measure of the effects of bisphenols; therefore, downstream analysis of transcripts and proteins crucial for proper early development provides more comprehensive information, which serves as more reliable indication of reproductive competence.

Quantification of Cx43 and 37 help solidify bisphenols’ mechanisms of action during oocyte maturation, thereby supporting BPA and its analogs’ effects on oocyte competence. Although there are limited existing studies investigating the roles of Cxs 37 and 43 in bovine oocytes, previous research has directly linked Cxs 37 and 43 to oocyte competence in mice. When genes encoding Cx37 are knocked out, folliculogenesis in ovaries is disrupted, and Cx37 null mutant oocytes suffer growth retardation, becoming unable to reach meiotic competence [54]. Compounds, such as BPA or BPS, affecting the expression of these connexins would, therefore, have direct consequences relating to improper oocyte maturation. BPA and BPS exposure had no significant effect on Cx43 mRNA expression (Figure 3A, Figure 4A,C); therefore, this connexin was removed from subsequent analyses. Changes in Cx37 mRNA expression, although not significant, were observed in COCs following bisphenol exposure. A more detailed analysis conducted only in denuded oocytes versus cumulus cells to understand exactly at which cellular level bisphenols had an effect, determined that BPA and BPS effects on Cx37 did not occur in the oocyte per se, but in the surrounding environment, the cumulus cells. Cx37 mRNA levels are significantly elevated in cumulus cells following bisphenols exposure. A study by Acuna-Hernandez et al. [4] that looked at BPA exposure in mice COCs using immunofluorescence found that BPA disrupts gap junctional intercellular communication (GJIC). They suggested that the altered GJIC, regulated by multiple Connexins, including Cx37, likely led to accelerated meiotic cycles and improper prophase I to metaphase II transition, resulting in poor bidirectional communication between oocytes and cumulus cells.

Further experiments at the protein levels showed that, while qPCR analysis demonstrated detectable levels of Cx37 mRNA expression, Cx37 protein was not detectable in either the oocytes or the cumulus cells. This could be due to a lack of translation of the mRNA within the oocyte. On the other hand, in COCs, exposure to BPA led to a significant increase in Cx37 protein expression, while BPS had no significant effect (Figure 5). This result is in contrast to the lack of effect seen in previous qPCR analysis. It can be speculated that BPA favors translation of Cx37 mRNA, and that all of the Cx37 mRNA is converted to protein. As a result, we see an increase in protein expression in COCs without a corresponding increase in transcripts’ expression.

Confocal microscopy was used to investigate whether bisphenols cause a change in localization of Cx37 protein within the COCs, that could be partially responsible for the endocrine disrupting effects. No visible localization changes were able to be detected among treatment groups (Figure 6). Cx37 was confirmed to be at the periphery of the oocyte and in the surrounding cumulus cells [33]. Lack of localization changes highlight the importance of change in quantity of the protein versus conformational changes.

Parallel experiments in the lab investigating changes in microRNA expression and methylation effectors within the oocyte and cumulus cells [38] suggest that epigenetic modifications could be linked to changes in connexin expression within gap junctions. MicroRNAs are epigenetic regulators, whose differential expression is involved in various stages of female reproduction. Our studies showed significant dysregulation of a number of miRNAs (miR-21, miR-155, and miR-34c) upon treatment of oocytes with BPA, presenting another mechanism of action potentially linked to connexins as well [38]. There is mounting evidence of a functional link between crucial connexins and microRNAs [55,56,57]. miRNAs are capable of regulating connexin expression levels. Caledron and Retamal [58] extensively review Cx-miRNA interactions in various systems and present an alternative pathway that contribute to the observed phenotypes. A study in porcine cumulus cells reported that over expression of miR-378 decreased Cx43 and Cx37 in cumulus cells and oocytes, respectively, resulting in interrupted oocyte maturation [59]. On the other hand, Cx expression levels can regulate miRNA expression. A study in mice cells and bones deficient in Cx43 found significantly reduced levels of miR-21 sufficient to induce cellular apoptosis [55].

The incorrect establishment of gap junctions could be one of the possible mechanisms through which bisphenols elicit their effects. The possibility that BPS or BPA affect Cx37 expression in cumulus cells could lead to alteration in other connexins and cell-to-cell communication, which may consequently result in decreased oocyte growth and oogenesis [29]. This localized effect in the cumulus cells may provide further insight into the mechanistic function of bisphenols. For this reason, cumulus cells were cultured in vitro in order to determine possible bisphenol effects on isolated cumulus cells. Exposure of cumulus cells to BPA led to a significant increase in Cx37 mRNA and protein expression, while BPS shows no significant effect (Figure 7 and Figure 8). This result confirms that BPA has significant effects on Cx37 channels in cumulus cells, thereby altering gap junction function, potentially impacting oocyte maturation, and contributing to reproductive dysfunction. The lack of effect on Cx37 expression in BPS-exposed cells suggests that the mechanism by which BPS affects cumulus cells does not involve the Cx37 subunit, and this bisphenol might act through a different mechanism or might not have significant effects on the oocyte surrounding environment and consequently on oocyte competence. This latter hypothesis would make BPS a better candidate than BPA to use in plastics. However, further investigation of other bisphenols that impact cumulus cells, such as BPF, is required to fully understand the possible involvement of these intracellular communication proteins in exerting the effects of bisphenols.

Since BPA is a lipophilic compound, it possesses the ability to modify cell membrane structure, hence altering the functionality of its proteins and channels, including their gap junctions [60]. A 2015 study conducted by Oh supported this theory [61], suggesting that due to BPA’s molecular weight, the compound was able to enter the intracellular pore regions of the connexons and obstruct their ionic flow [61]. BPA is likely interfering with gap junction function of the cumulus cells by altering the cell membrane structure and fluidity [61]. Musil et al. [62] suggested that, although connexins have a high turnover rate, connexin degradation can be modulated to increase gap junction function. They found that proteasome inhibitors (PI) increase connexin availability for gap junction assembly by decreasing connexin turnover. The researchers proposed that PI exerted indirect effects on the connexins, including activating signaling events that increased gap junction formation [62]. Ge et al. showed that BPA downregulates proteasomes in Sertoli cells [63]. This suggests that BPA may act as a proteasome inhibitor and the increase in Cx37 protein expression resulted from a decreased turnover rate.

Conversely, no research has been found connecting the effects of BPS to proteasome downregulation, which may explain why it did not increase Cx37 protein expression in our study, yet we did see an increase in transcript levels in in vitro matured cumulus cells. BPA and BPS very likely have different mechanisms of action and further research is required before an accurate conclusion on BPS safety can be made [64,65,66]. Teteau et al. [12] and Amar et al. [22] investigated the mechanism by which BPS can affect sheep and human granulosa cells, respectively. They reported that BPS does not affect cell viability or proliferation, which may explain why we did not see an observable change in flask confluency in the BPS group. Nevertheless, these studies do report that BPS impairs steroidogenesis with disruptions in estradiol and progesterone secretions and dysregulated gene expression of crucial steroidogenic genes [12,22]. This strengthens our indication that BPS may work through a different mechanism than BPA. Moreover, studies have reported that crucial processes including steroidogenesis relies on the communication between oocytes and cumulus cells [67,68,69]. Therefore, we speculate that any disruptions on cell to cell communication may have downstream consequences on the basic functions of cumulus cells.

To our knowledge, the only studies that looked at the effects of BPA on Cx37 include one by Acuna Hernandez et al. [4] and another by Park et al. [70] in mice and porcine oocytes, respectively. Both studies reported disruptions in GJIC; however, one study reports no change in Cx37 protein or transcript expression [4] and the other reports a significant reduction in Cx37 mRNA [70]. The discrepancies between those studies and our findings could be related to differences in species, dose, or treatment time. Therefore, to our knowledge, this study is the first to report an increase in Cx37 mRNA and protein expression under the current LOAEL dose (0.05 mg/mL) [37] in bovine oocytes and cumulus cells treated for 24 h. Here, we speculate that the increase in expression can be as detrimental to GJIC and subsequently, the developmental capacity of oocytes.

Zhang et al. [71] found that testosterone significantly increased Cx37 protein expression in mice COCs. Therefore, BPA could be mimicking androgens, binding to the androgen receptor (AR) and eliciting this effect [72]. Zhang et al. [71] concluded that this effect promoted follicular growth rather than impeded it. Although BPA might be mimicking androgens, it is likely triggering a different response that, conversely, disrupts development. In porcine luminal epithelium cells, estradiol exposure also resulted in an increase in Cx37 expression [73]. The changes in connexin expression were significantly linked to apoptosis in vitro as well as cell differentiation [73]. Since BPA is also primarily an estrogen mimic, this could explain the similar results we see in both Cx37 expression as well as the reduction in confluent cumulus cells treated with BPA [10]. The mechanism of action employed by bisphenols to increase Cx37 expression remains to be elucidated as well as the exact disruptive downstream effects of this increased expression. It is speculated that increased Cx37 could increase GJIC and disrupt the activity of the proteins. This could lead to inappropriate shuffling of molecules within COCs that alter global gene expression and interfere with proper oocyte competence.

## 5. Conclusions

The results from this study demonstrate the significance impact of BPA and, to a lesser extent, of BPS on Cx37 in bovine cumulus cells, which is vital for the assembly of gap junctions that maintain the oocyte’s meiotic maturation. Neither BPA nor BPS caused significant variability to Cx43 expression for all sample groups or to Cx37 expression in oocyte and COC groups. BPS treatment did not produce a statistically significant difference from control or vehicle in the parameters analyzed. Further research should aim to determine the mechanism through which BPS acts on Cx37 and other connexin molecules and how their interference contributes to gap junction assembly and communication within the follicle. It remains a possibility that BPS holds a negative impact on oogenesis and embryo development although not comparable to the effects observed by BPA, therefore making it a better alternative to BPA.

As Cx37 is imperative to cell communication during development, BPA having an effect on this protein likely means it is also affecting oocyte–cumulus cells communication. This could lead to an inability of affected oocytes to produce a viable embryo. Analysis of GJIC within COCs and cumulus cells via oocyte immunofluorescence after varying dosages of bisphenols would be extremely informative, especially when compared to the results of BPA-treated COCs. This would not only provide more information on the impact of bisphenols on reproductive health, but also on the role of Connexin molecules in reproductive capabilities.

## Figures and Tables

**Table 1:** Primers used in qPCR.

Gene	GenBank Accession #	Source	Primer Sequence (5′-3′)	IVM Primer Efficiency (%)	IVC Primer Efficiency (%)	Product Size (bp)
**GAPDH**	NM_001034034.1	[39]	5′-ttcctggtacgacaatgaatttg-3′ 5′-ggagatggggcaggactc-3′	102.3	100.8	153
**PPIA**	NM_178320.2	[10]	5′-tcttgtccatggcaaatgctg-3′ 5′-tttcacctgccaaagtaccac-3′	98.8	99.0	111
**Cx37**	NM_001083738.1	[28]	5′-gactcatctccctggtgctc-3′ 5′-gttctgctcactggacgaca-3′	97.0	100.3	221
**Cx43**	NM_174068	[40]	5′-gtcttcgaggtggccttcttg-3′ 5′-agtccacctgatgtgggcag-3′	101.9	NA	104

## Data Availability

Not Applicable.
